# The epidemiology of primary FSGS including cluster analysis over a 20-year period

**DOI:** 10.1186/s12882-023-03405-w

**Published:** 2023-12-10

**Authors:** Thomas McDonnell, Joshua Storrar, Rajkumar Chinnadurai, Calvin Heal, Constantina Chrysochou, James Ritchie, Francesco Rainone, Dimitrios Poulikakos, Philip Kalra, Smeeta Sinha

**Affiliations:** 1 Donal O’Donoghue Renal Research Centre, Northern Care Alliance NHS Foundation Trust, Salford Royal Hospital, Stott Lane, Salford, UK; 2 Faculty of Biology, Medicine and Health, School of Medical Sciences, University of Manchester, Oxford Road, Manchester, UK; 3 Centre for Biostatistics, University of Manchester, Oxford Road, Manchester, UK

**Keywords:** Cluster analysis, Epidemiology, FSGS, Immunosuppression

## Abstract

**Introduction:**

Focal segmental glomerulosclerosis (FSGS) is one of the leading causes of nephrotic syndrome in adults. This epidemiological study describes a renal centre’s 20-year experience of primary FSGS.

**Methods:**

Patients were identified with a diagnosis of primary FSGS after exclusion of known secondary causes. In this retrospective observational study, data was collected for baseline demographics, immunosuppression and outcomes. A two-step cluster analysis was used to identify natural groupings within the dataset.

**Results:**

The total cohort was made up of 87 patients. Those who received immunosuppression had lower median serum albumin than those who did not- 23g/L vs 40g/L (*p*<0.001) and higher median urine protein creatinine ratios (uPCR)- 795mg/mmol vs 318mg/mmol (*p* <0.001). They were more likely to achieve complete remission (62% vs 40%, *p*=0.041), but relapsed more 48.6% vs 22% (*p*=0.027). Overall 5 year mortality was 10.3% and 5 year progression to RRT was seen in 17.2%. Complete remission was observed in 49.4%. The 2-step cluster analysis separated the cohort into 3 clusters: cluster 1 (*n*=26) with ‘nephrotic-range proteinuria’; cluster 2 (*n*=43) with ‘non-nephrotic-range proteinuria’; and cluster 3 (*n*=18) with nephrotic syndrome. Immunosuppression use was comparable in clusters 1 and 3, but lower in cluster 2 (77.8% and 69.2% vs 11.6%, *p*<0.001). Rates of complete remission were greatest in clusters 1 and 3 vs cluster 2: 57.7% and 66.7% vs 37.2%.

**Conclusion:**

People who received immunosuppression had lower serum albumin and achieved remission more frequently, but were also prone to relapse. Our cluster analysis highlighted 3 FSGS phenotypes: a nephrotic cluster that clearly require immunosuppression; a cohort with preserved serum albumin and non-nephrotic range proteinuria who will benefit from supportive care; and lastly a cluster with heavy proteinuria but serum albumin > 30g/L. This group may still have immune mediated disease and thus could potentially benefit from immunosuppression.

**Trial registration:**

This study protocol was reviewed and approved by the ‘Research and Innovation committee of the Northern Care Alliance NHS Group’, study approval number (Ref: ID 22HIP54).

**Supplementary Information:**

The online version contains supplementary material available at 10.1186/s12882-023-03405-w.

## Background

Focal segmental glomerulosclerosis (FSGS) is one of the leading causes of nephrotic syndrome (NS) in adults. It refers to a histological pattern on kidney biopsy consisting of glomerular sclerosis and podocyte effacement. This can be the result of both a primary disease process or secondary to other causes, such as drugs, autoimmune diseases, or infections [1]. Historically it has been categorised based on findings on light microscopy; the Columbia classification outlines 5 distinct histologic variants [2] but it does not differentiate between primary and secondary causes, and outside of the presence of the steroid responsive ‘tip variant’ or the aggressive ‘collapsing variant’, it is limited in its prognostication [3]. As a result, there has been a move away from a morphological categorisation and current guidelines recommend aetiology-based classification [1]. Once the histopathological lesion is found, FSGS can be subcategorised into primary, secondary, genetic or of undetermined cause (UC) [4].

Primary FSGS is defined by the presence of NS: proteinuria >3.5g/d, hypoalbuminemia (<30g/L) and oedema which is usually abrupt in onset. It requires the exclusion of known secondary causes, including genetic causes, and treatment with immunosuppression (IS) is recommended. Although the aetiology of primary FSGS is unknown, it is widely hypothesised that it is due to an undiscovered circulating permeability factor [1]. This theory is supported by the rapid recurrence of FSGS post kidney transplantation and the reduction of proteinuria seen with IS [5, 6].

Secondary FSGS, by contrast, often presents with varying degrees of proteinuria and preserved serum albumin (sAlb), which is insidious in onset. Causes can be subclassified into viral, drug induced, autoimmune and ‘adaptive’ FSGS (from glomerular hyperfiltration). In secondary FSGS treatment is directed at the underlying cause and/or is supportive, including the use of renin-angiotensin system (RAS) blockade and blood pressure control. Immunosuppression is unlikely to be beneficial due to the absence of the putative circulating factor. A number of genetic causes of FSGS have also been identified [7].

There are a number of recent US [8–11] cohorts which describe rising prevalence of FSGS over the last 4 decades with associated increased rates of end-stage kidney disease (ESKD). Despite this there is less published data on rates of FSGS in the UK. One study in Northern Ireland reported a much lower rate of FSGS [12] is in keeping with other European studies [13–15]. This epidemiological study aims to describe a cohort of patients with primary FSGS diagnosed over a 20-year period at a tertiary renal centre in the UK. The goal was firstly to describe the whole cohort, and secondly to identify clusters (in a similar manner to that suggested by KDIGO) and undertake survival analysis to aid with treatment decisions.

## Methods

This was a retrospective observational longitudinal study conducted on patients diagnosed with primary FSGS at a tertiary renal centre (Salford Royal Hospital, UK), encompassing a catchment population of 1.55 million, over a period of 2 decades.

**Fig. 1:**
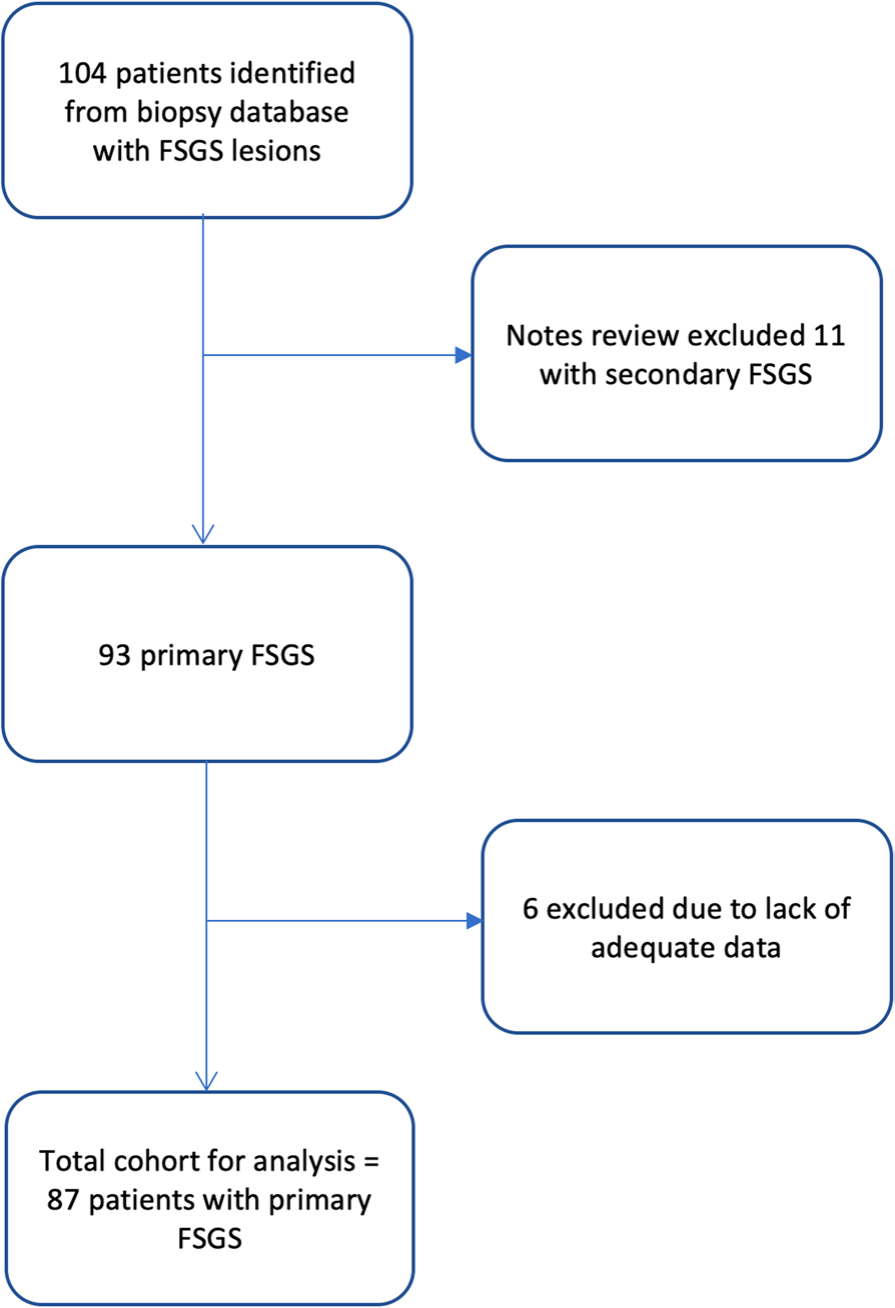
Flowchart of patient recruitment to study

The Salford kidney biopsy database was screened for patients with an FSGS lesion seen on light microscopy between January 2000 and December 2019. Figure 1 shows the flow-chart for study inclusion. Initially 104 patients were identified, however 11 were excluded due to the identification of a secondary cause: x3 hypertensive, x2 hyperfiltration and x1 renovascular disease, anabolic steroid use, previous episode of meningococcal septicaemia, paraneoplastic, chronic lymphoid leukemia and prior pre-eclampsia. A further 6 were excluded due to inadequate data for meaningful evaluation, resulting in 87 patients for analysis.

Date of kidney biopsy was used as study baseline. All patients were treatment naïve at baseline. Study endpoint was either date of commencement of renal replacement therapy (RRT), death, end of analysis period (31/05/2021) or last clinic appointment.

Baseline characteristics, laboratory results, angiotensin converting enzyme inhibitors (ACEi) or angiotensin receptor blockers (ARBs) and immunosuppression use, date of initiation of RRT (either transplantation or dialysis), mortality and relapse/remission rates were collected from the electronic patient record (EPR). All baseline characteristics and laboratory results were obtained within 6 months from the time of biopsy. There was no departmental protocol in place for immunosuppression treatment of FSGS; choice was based on a combination of individual clinician preference and international guidance. Kidney Disease Improving Global Outcomes (KDIGO) guidelines have been followed since 2012.

Hypertension was defined as hypertension recorded in EPR, and/or receiving antihypertensive therapy. A comorbidity of cardiovascular disease (CVD) included a history of ischaemic heart disease, heart failure, cerebrovascular disease, or peripheral vascular disease.

Aligning with KDIGO definitions [4], complete remission from NS was defined as a urine protein creatinine ratio [uPCR] <30mg/mmol, stable serum creatinine and sAlb >35g/L; partial remission was defined as a reduction in proteinuria to uPCR 30-350mg/mmol and a decrease >50% from baseline. Combined remission is the sum of partial and complete remission. Relapse was defined as a uPCR >350mg/mmol after complete remission or an increase in proteinuria by >50% during partial remission.

### Statistical methodology

Continuous variables that were non-normally distributed were presented as median (interquartile range) with a *p*-value by Mann Whitney-U. If normally distributed, data was presented as mean +/- standard deviation with a *p*-value by T-test. Categorical values were presented as number (percentage) with a *p*-value by Chi-squared test.

A two-step cluster analysis was used to reveal natural groupings (clusters) within the dataset [16]. Pre-specified variables that contributed to the clustering were: sAlb, uPCR and estimated glomerular filtration rate (eGFR) at time of biopsy. Here ‘natural groupings' refer to clusters formed by the two-step cluster analysis algorithm based on these pre-specified variables, which captures inherent similarities or patterns within the dataset without imposing any predefined criteria or assumptions.

All statistical analysis was performed using IBM SPSS (version 25, licensed to the University of Manchester).

## Results

Baseline characteristics, laboratory results and outcomes for the total cohort are presented in Table 1. Mean age for the cohort was 49.3 years (+/-17.9 years), 53 (60.9%) were male and 75 (86.2%) were White. Pre-existing diabetes (all type 2) was present in 8 (9.2%), hypertension in 46 (52.9%) and CVD in 13 (15.1%). At the time of biopsy, the median blood pressure was 130/79 mmHg, creatinine 135µmol/L (1.53mg/dL), eGFR 46ml/min/1.73m^2^, uPCR 573mg/mmol and sAlb 33g/L. Nephrotic syndrome was present in 29 (33.3%). Partial, complete, and combined remission rates were 20 (23%), 43 (49.4%) and 63 (72.4%) respectively. Median time to partial or complete remission was 523 days. Relapse occurred in 29 (33.7%). ACEi/ARB use was seen in 79%, with immunosuppression used in 42.5%. Progression to RRT was observed in 24 (27.6%), and the 5- and 10-year RRT rate was 17.2% and 25.3% respectively. Overall mortality was 24 (27.6%), and the 5- and 10-year mortality rate was 10.3% and 19.5% respectively. Median follow-up duration was 91 months (39 – 129), 14 patients were either discharged from clinic or losts to follow-up. The incidence rate based on our catchment population of 1.55 million was 2.81/million/year.

**Table 1:** Baseline demographics, laboratory values, selected treatment and outcomes for the total cohort, those who received immunosuppression and those who did not

Variable		Total cohort (*n*=87)	Immunosuppression (*n*= 37)	No immunosuppression (*n*= 50)	*P*- value
Age		49.3 (+/- 17.9)	52.7 (+/- 18.7)	46.7 (+/- 17.0)	0.118
Male		53 (60.9)	24 (64.9)	29 (58)	0.516
White ethnicity		75 (86.2)	33 (89.2)	42 (84)	0.488
Diabetes		8 (9.2)	3 (8.1)	5 (10)	0.651
Hypertension		46 (52.9)	19 (51.4)	27 (54)	0.648
Cardiovascular disease		13 (15.1)	3 (8.1%)	10 (20)	0.124
Systolic BP at biopsy, mmHg		130 (120- 140)	130 (120 -140)	128 (119 – 135)	0.627
Diastolic BP at biopsy, mmHg		79.3 (11.5)	80.1 (+/-13.2)	78.7 (+/-10.0)	0.594
Creatinine, µmol/L		135 (90-218)	134 (96.8 – 227)	134 (88 – 201)	0.874
eGFR, ml/min/1.73m^2^		46 (27 – 76)	42.5 (25 – 71.3)	46 (28.5 – 70)	0.874
uPCR, mg/mmol		573 (210 – 811)	795 (627 – 998)	318 (193 – 692)	**<0.001**
Haemoglobin, g/L		124 (110 – 144)	118 (104 – 132)	125 (110 – 153)	**0.012**
Corrected calcium, mmol/L		2.32 (2.24 – 2.42)	2.30 (2.22 -0 2.39)	2.37 (2.30 – 2.46)	0.26
Phosphate, mmol/L		1.23 (1.05 – 1.37)	1.30 (1.17 – 1.39)	1.17 (1.04 – 1.33)	**0.039**
Albumin, g/L		33 (23-41)	23 (19.5 – 29.3)	40 (33 – 43)	**<0.001**
Presented with nephrotic syndrome		29 (33.3)	25 (67.6)	4 (4.6)	**<0.001**
Remission	Partial	20 (23)	8 (21.6)	12 (24)	0.794
	Complete	43 (49.4)	23 (62.2)	20 (40)	**0.041**
	Combined	63 (72.4)	31 (83.8)	32 (64)	**0.041**
Time to remission (days)		523 (159-1231)	191 (103.5 – 598)	806 (255 – 1677)	**0.001**
Relapse		29 (33.7)	18 (48.6)	11 (22)	**0.027**
ACEi/ ARB		67 (79)	28 (75.7)	39 (78)	0.799
Immunosuppression		37 (42.5)	-	-	-
RRT^a^	Total	24 (27.6)	10 (27)	14 (28)	0.920
	5 year	15 (17.2)	7(18.9)	8 (16)	0.722
	10 year	22 (25.3)	9 (24.3)	13 (26)	0.859
Mortality^a^	Total	24 (27.6)	10 (27)	14 (28)	0.920
	5 year	9 (10.3)	5 (13.5)	4 (8)	0.404
	10 year	17 (19.5)	6 (16.2)	11 (22)	0.501
Follow up duration, months		91 (39 – 129)	99 (37 – 137)	108 (57.5 – 140)	0.810

*ACEi* Angiotensin converting enzyme inhibitor, *ARB* Angiotensin receptor blocker, *BP* Blood pressure, *eGFR* Estimated glomerular filtration rate, *RRT* Renal replacement therapy, *uPCR* urine protein creatinine ratio

Continuous variables presented as median (interquartile range), unless normally distributed when presented as mean +/- standard deviation, *p*-value by Mann Whitney-U or ANOVA test. Categorical values presented as number (percentage), *p*-value by Chi-squared test

^a^5 and 10-year RRT and Mortality rates are cumulative, 5 year mortality and RRT rates are therefore included within 10 year mortality and RRT rates

Table 1 also compares the 37 subjects who received immunosuppression with the 50 who did not. Those who received immunosuppression had a lower sAlb (23g/L vs 40g/L, *p*< 0.001), higher uPCR (795mg/mmol vs 318mg/mmol, *p* <0.001) and Phosphate (1.3mmol/L vs 1.17mmol/L, *p*=0.039) and a lower Hb (118g/L vs 125g/L, *p*=0.001). The immunosuppression group were more likely to achieve complete (62% vs 40%, *p*=0.041) and combined remission (84% vs 64%, *p*=0.041). The time to reach remission in the immunosuppressed group was shorter (191 vs 806 days, *p*=0.001), however they were more likely to relapse (49% vs 22%, *p*=0.027). In the immunosuppression group, 25 (67.6%) presented with nephrotic syndrome, vs 4 (4.6%) in those who did not receive immunosuppression, *p*<0.001.

Frequency and type of immunosuppression used can be seen in Table 2. Prednisolone alone was used in 19 subjects (51% of those given immunosuppression). A combination of prednisolone with either one or two other agents was used in 16 (43.2%) and mycophenolate mofetil (MMF) was used as a single agent in 2 (5.5%) subjects.

**Table 2:** Types of immunosuppression used within first year of immunosuppression use

**Immunosuppression type**		**Frequency *n*=37**
Prednisolone		19 (51%)
Prednisolone +	Ciclosporin	9 (24%)
	Tacrolimus	4 (11%)
	MMF	2 (5.5%)
	Ciclosporin + MMF	1 (3%)
	**Total**	**16**
Mycophenolate mofetil (MMF)		2 (5.5%)

Immunosuppression recorded is that which was started within the first 12 months of immunosuppression use

**Fig. 2:**
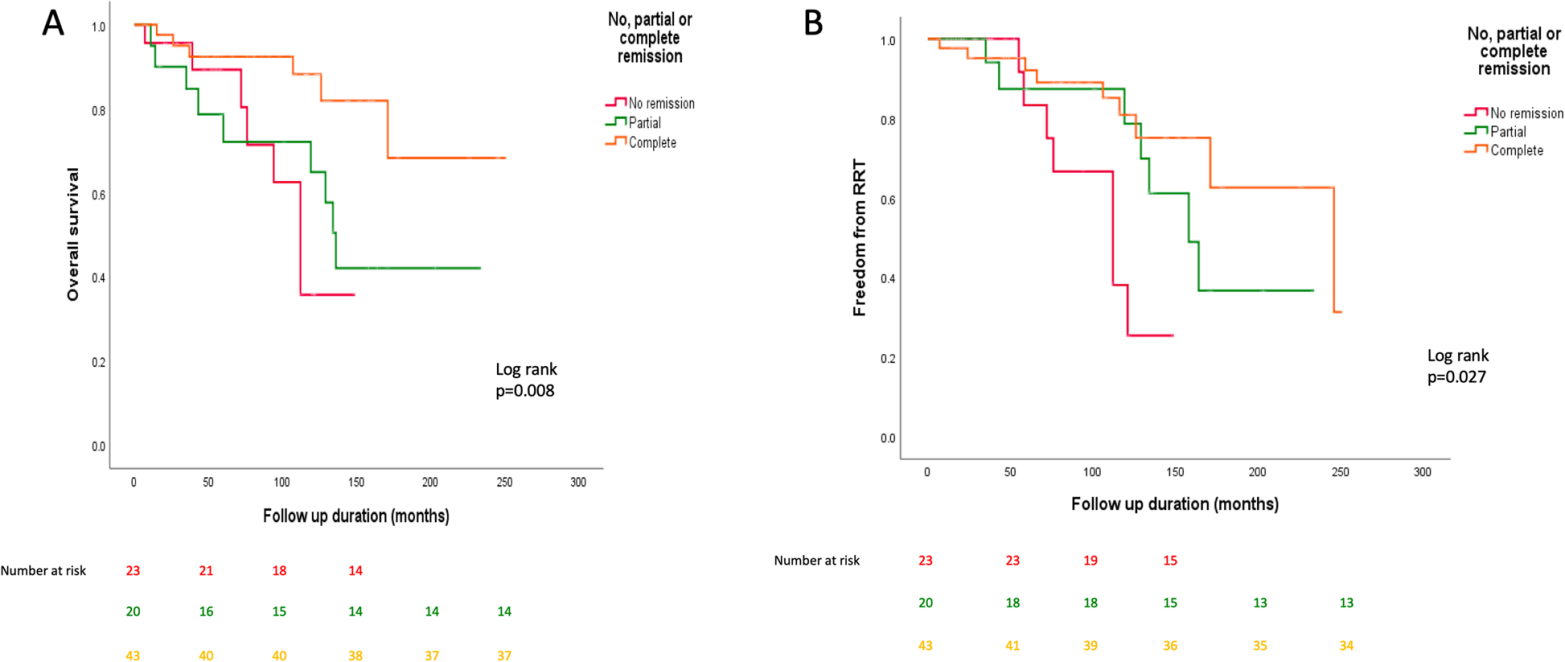
KM curves showing differences in survival and freedom from RRT by remission status (no, partial or complete remission)

The effect of remission (none, partial or complete) on overall survival and freedom from RRT was assessed using Kaplan-Meier curves (shown in Fig. 2). Those who reached complete remission had a better overall survival than those who achieved partial or no remission (*p*=0.008). Those who achieved complete or partial remission were less likely to progress to RRT (*p*=0.027).

**Fig. 3:**
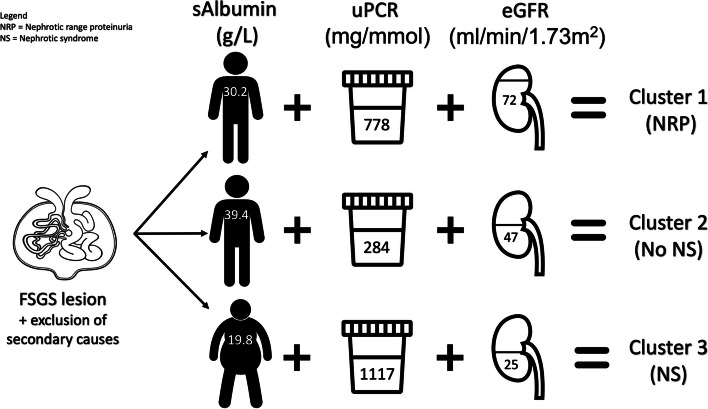
Graphical representation of 3 clusters

A two-step cluster analysis was undertaken to reveal *natural* groups (see methods) within the dataset based on the inputted variables (shown in Fig. 3). sAlb, uPCR and eGFR at presentation were selected as the inputted variables as these are the factors felt to affect treatment decisions most significantly. Three naturally occurring clusters/phenotypes within this primary FSGS dataset were identified by the algorithm: cluster 1 (26 patients), cluster 2 (43 patients) and cluster 3 (18 patients). The cluster ratio of the largest cluster to smallest cluster was 2.39 (a ratio of less than 3 is acceptable for cluster sizing). sAlb was the most important variable contributing to the cluster group as per the algorithm, followed by uPCR and finally eGFR.

Two-step cluster analysis presents variables as mean averages. The 18 patients in cluster 3 were those with highest protein excretion (mean uPCR at presentation was 1117 mg/mmol) and lowest sAlb (19.8 g/L) as well as lowest eGFR (25.3ml/min/1.73m^2^). This cluster represents a heavily nephrotic patient cohort. The 43 patients in cluster 2 had the lowest protein excretion (uPCR 284 mg/mmol), highest sAlb (39.4 g/L) and a mean eGFR of 46.4 ml/min/1.73m^2^. This cluster represents the non-nephrotic patients in the cohort. The 26 patients in cluster 1 had a sAlb of 30.2 g/L, a uPCR of 778 mg/mmol and the highest eGFR (71.7 ml/min/1.73m^2^). This cluster represents those patients with nephrotic range proteinuria (NRP).

Table 3 depicts the baseline characteristics and outcome data for the three clusters. Cluster 3, the ‘nephrotic’ group, were the oldest (mean age  60.5 years) and had the highest blood pressure (median 132/78mmHg). They had the greatest use of immunosuppression (77.8%), greatest complete remission rate (66.7%) and highest overall mortality (44.4%). We also calculated 5- and 10- year mortality rates given the long follow up duration for the study. Whilst there was no statistically significant differences between the 3 clusters, there was a trend towards increasing rates for cluster 3.

**Table 3:** Total cohort split according to 3 clusters

**Variable**		**Cluster 1 (*n*= 26)**	**Cluster 2 (*n*= 43)**	**Cluster 3 (*n*= 18)**	***P*-value**
Age		47.0 (+/- 15.4)	43.5 (+/- 12.1)	60.5 (+/- 16.4)	**0.004**
Male		15 (57.7)	28 (65.1)	10 (55.6)	0.723
White ethnicity		20 (76.9)	39 (90.7)	16 (88.9)	0.256
Diabetes		3 (12)	5 (11.6)	0 (0)	0.311
Hypertension		12 (48)	26 (60.5)	8 (44.4)	0.420
Cardiovascular disease		3 (11.5)	8 (18.6)	2 (11.1)	0.638
Systolic BP at biopsy, mmHg		122 (116.8 – 130)	130 (120 – 142.3)	132 (120-140)	**0.018**
Diastolic BP at biopsy, mmHg		75.7 (+/- 9.5)	81.2 (+/-12.0)	78.1 (+/-12.1)	**0.005**
Creatinine, µmol/L		93.5 (63.3 – 110.5)	139.5 (119.3 – 222)	226 (166 – 272)	**<0.001**
eGFR, ml/min/1.73m^2^		80 (52 – 90)	38.5 (27.3 – 58)	22 (16 – 39)	**<0.001**
uPCR, mg/mmol		782 (617 – 930.3)	227 (173.3 – 373)	852 (776 – 1456)	**<0.001**
Haemoglobin, g/L		122 (105.5 – 134)	127 (108.8 – 157.8)	113 (106 – 120)	**0.002**
Corrected calcium, mmol/L		2.31 (2.23 – 2.43)	2.37 (2.28 – 2.45)	2.31 (2.23 – 2.36)	0.664
Phosphate, mmol/L		1.25 (1.15 – 1.35)	1.14 (0.98 – 1.33)	1.35 (1.24 – 1.74)	**0.01**
Albumin at biopsy, g/L		29 (24.3 – 34.5)	42 (37.3 – 43.8)	20 (17- 22)	**<0.001**
Immunosuppression		18 (69.2)	5 (11.6)	14 (77.8)	**<0.001**
Albumin at TOI, g/L		27 (21 -33)	30 (26.5 – 40.5)	18 (17 – 23.5)	**0.003**
Presented with nephrotic syndrome		12 (41.4)	0 (0)	17 (94.4)	**<0.001**
Remission	Partial	6 (23.1)	11 (25.6)	3 (16.7)	0.752
	Complete	15 (57.7)	16 (37.2)	12 (66.7)	0.067
	Combined	21 (80.8)	27 (62.8)	15 (83.3)	0.137
Time to remission, days		288.5 (147 – 734.5)	807 (196.5 – 1880)	195 (76 – 612)	**0.006**
Relapse		13 (50.0)	9 (21.4)	7 (38.9)	**0.046**
ACEi/ ARB		22 (84.6)	36 (83.7)	9 (50)	**0.009**
RRT^a^	Total	5 (19.2)	13 (30.2)	6 (33.3)	0.507
	5 year	3 (11.5)	6 (14)	6 (33.3)	0.123
	10 year	4 (15.4)	12 (27.9)	6 (33.3)	0.346
Mortality^a^	Total	3 (11.5)	13 (30.2)	8 (44.4)	**0.048**
	5 year	2 (7.7)	3 (7.0)	4 (22.2)	0.177
	10 year	2 (7.7)	10 (23.3)	5 (27.8)	0.176
Follow up duration, months		111 (54.5 – 147.5)	112 (47.3 – 142)	86 (19 – 129)	0.532

*ACEi* Angiotensin converting enzyme inhibitor, *ARB* Angiotensin receptor blocker, *BP* Blood pressure, *eGFR* Estimated glomerular filtration rate, *RRT* Renal replacement therapy, *TOI* time of immunosuppression, *uPCR* Urine protein creatinine ratio

Continuous variables presented as median (interquartile range), unless normally distributed when presented as mean +/- standard deviation, *p*-value by Mann Whitney-U or ANOVA test. Categorical values presented as number (percentage), *p*-value by Chi-squared test

^a^5 and 10-year RRT and Mortality rates are cumulative, 5-year mortality and RRT rates are therefore included within 10 year mortality and RRT rates

Cluster 2, the ‘non-nephrotic’ group, had the lowest rates of immunosuppression use (11.6%), lowest rates of complete (37.2%) and combined remission (62.8%), as well as taking the longest to achieve remission (median 807 days), though their relapse rates were the lowest (21.4%). RRT rates (30.2%) were comparable to cluster 3 (the ‘nephrotic cohort’).

Cluster 1, the group with ‘nephrotic range proteinuria’, had the lowest blood pressure (median 122/76mmHg). Rates of immunosuppression (69.2%) and combined partial and complete remission (80.8%) were comparable to cluster 3 (the nephrotic cohort). Relapse rates were highest (50%) but rates of RRT (19.2%) and mortality (11.5%) were lowest.

**Fig. 4:**
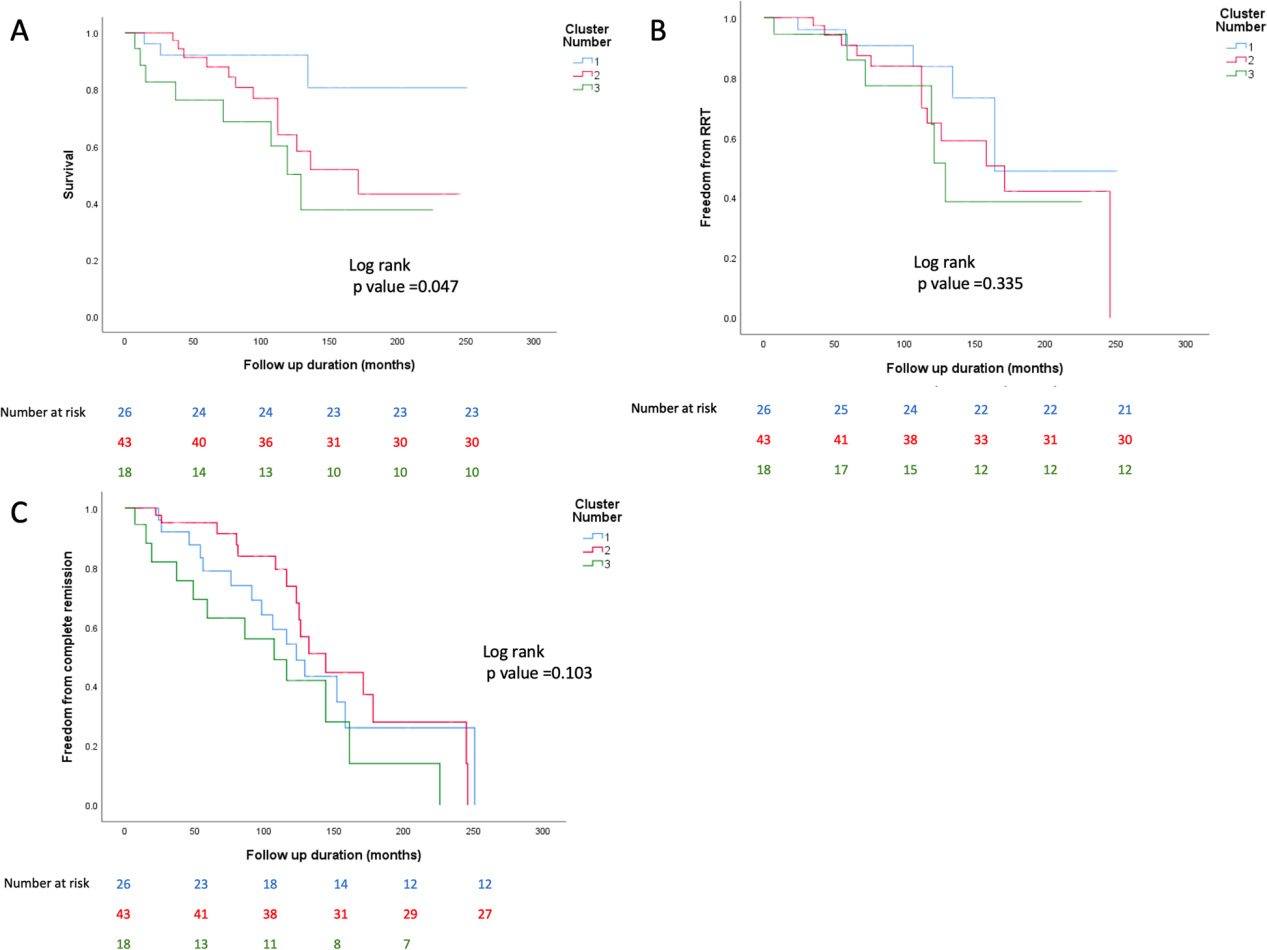
KM curves showing differences in survival, freedom from RRT and complete remission rates amongst the 3 clusters

Figure 4 shows Kaplan-Meier curves for survival (A), freedom from RRT (B) and complete remission rates (C) amongst the three clusters. Only the survival curve demonstrates a significant difference between the 3 groups (log rank *p*=0.047).

## Discussion

The combined lack of RCTs guiding treatment and the inability to measure the putative permeability factor makes the diagnosis and treatment of primary FSGS a challenge. This study provides a real-world insight into patients with FSGS lesions on biopsy, after exclusion of secondary causes, over the last 20 years and identifies phenotypes which can guide treatment decisions and aid prognostication.

Over the last 10 years there have been 3 US epidemiological reviews of FSGS which provide information on treatment and outcomes [17–19], in addition to an older Dutch study [20] and three reviews from Asia [21–23]. However there have been no studies specifically detailing the epidemiology and outcomes of FSGS in a UK population.

The baseline characteristics and renal parameters seen in this study are similar to the published literature. Only Jafry et al. [22] had a younger cohort. Forster et al. [18] and Jafry et al. had a much higher proportion of males in their cohorts. Jafry et al. was the only study in which there was a clear nephrotic cohort with an average sAlb of 21g/L but a similar degree of proteinuria to our study. Most of the studies reported an average sAlb of 33 g/l and even when Hommos et al. split their cohort based on greater than 80% foot process effacement, the sAlb remained at 33g/L. Each study comments variably on outcome data, however rates of immunosuppression are comparable to our study in those where it was reported [18, 20, 21]. Rates of ESKD in our study (27.6%) were comparable to Forster et al. but lower than Deegens et al (37%). Kwon et al. [21] reported low rates of ESKD at 8%, however their follow-up period was very short at 34.5 months (for comparison our 5-year RRT rate was 17.2%). Rates of complete remission varied significantly: Forster and Kwon had lower rates of 26%, whilst Deegans had similar rates to our cohort (40%), and Jafry had the highest rates (62%)- interestingly this was the nephrotic cohort.

Kawaguchi et al. [23] assessed histological FSGS lesions in a Japanese cohort of 304 patients between 2010 and 2013 but found no significant difference in outcomes between the different lesions (tip, perihilar, cellular, collapsing and not otherwise specified) which supports the more contemporary aetiology-based classification. They did demonstrate that proteinuria remission was associated with improved outcomes, which is also demonstrated in our study (see Fig. 2). In their cohort, 55% received immunosuppression, in comparison to 42.5% in our cohort, and 45% achieved complete remission, similar to our cohort (49.4%).

When reviewing our dataset, the median sAlb and uPCR was 33g/L (23-42g/L) and 573mg/mmol (210-811mg/mmol) respectively, which is similar to the above referenced studies. However, the interquartile ranges (IQRs) demonstrate that there is large variability within the sample suggesting that there may be different phenotypic presentations within this cohort.

A 2-step clustering algorithm produced natural groupings based on the inputted variables: sAlb, uPCR and eGFR. The 2-step cluster produced three clusters (shown in Fig. 3) Cluster 3 (*n*=18) were nephrotic and can be considered classical ‘primary FSGS’. The management of these patients is straightforward and international guidance endorses immunosuppression with prednisolone, and a high proportion were immunosuppressed [4]. Cluster 2 (*n*=43) represents a cohort with non-nephrotic range proteinuria. This cohort is likely to represent an FSGS-UC phenotype rather than a true ‘primary/autoimmune/ antibody driven’ FSGS and genetic testing should be considered in this group [6]. There were no increased rates of hypertension, CVD, or diabetes in cluster 2 to suggest this was adaptive FSGS however the group were not assessed for obesity or prematurity so this may still have represented an adaptive cohort. Immunosuppression is not recommended in this group and indeed only a small proportion (11.6%) received immunosuppression.

The last group, cluster 1 (*n*=26), represented those in the cohort with nephrotic range proteinuria without ‘full blown’ nephrotic syndrome (normal sAlb). This cluster represents the most interesting phenotype from a treatment perspective as they would not be considered ‘primary FSGS’ by KDIGO classification (as they do not have NS) and thus immunosuppression would not be recommended. Despite this, cluster 1 is clearly a separate group to cluster 2 and interestingly, rates of immunosuppression were high in this cluster and comparable to the nephrotic cluster 3.

Some outcomes appeared to be similar between cluster 1 (nephrotic range proteinuria group) and cluster 3 (nephrotic syndrome group) (see Table 3), including complete remission (57.7% in cluster 1 and 66.7% in cluster 3- similar to Jafry et al’s. nephrotic cohort) and time to remission, which was shorter in clusters 1 and 3. There were no differences in RRT rates between clusters 1 and 3, however cluster 1 had a lower mortality rate (11.5% vs 44.4%); although this could be explained by cluster 1 being the youngest.

Caution must be applied when drawing conclusions from the outcome variables of these three clusters due to their small sample size and the retrospective nature of the analysis. However, whereas cluster 2 is most probably an adaptive/ FSGS- UC cohort and unlikely to represent primary FSGS, cluster 1 may represent a primary FSGS phenotype, despite not meeting KDIGO criteria for this.

The diagnosis of primary FSGS is challenging: the concept of ‘primary FSGS’ is one of an autoimmune podocytopathy. KDIGO suggest the diagnosis of primary FSGS should only be made in the presence of NS, where NS is used as a surrogate marker for a measurable permeability factor. However, it may be that NS is not sensitive enough to capture all primary FSGS. In this study, the average sAlb was 33 g/L with nephrotic syndrome present in only 33% at time of biopsy. Of those who received immunosuppression only 67.6% were nephrotic (thus 32.4% were not). Within all of the above-referenced epidemiological reviews of primary FSGS only one (Jafry et al.) included a cohort with an *average* sAlb <30 g/L. Rates of NS in studies including primary FSGS can vary significantly, between 54-90% [6]. This may be due to the inclusion of unrecognised adult genetic or otherwise secondary forms of FSGS. However, there may be varying degrees of hypoalbuminemia and proteinuria seen in those at different stages of primary FSGS, similar to the varying degrees of proteinuria with varying levels of anti-phospholipase A2 receptor antibodies (anti-PLA2R) in primary membranous nephropathy [24]. If this is so, the presence of NS alone *may* be too blunt a tool for diagnosis in primary FSGS.

Cluster 1 (nephrotic range proteinuria but preserved serum albumin) could still represent a permeability factor/ antibody driven disease that is either earlier in its presentation or with an antibody at lower titre. Indeed, sAlb at time of immunosuppression was 2g/L lower in both cluster 1 and 3 and highlights the progressive nature of primary FSGS and the need for close follow-up before immunosuppression. These clusters also highlight the difficulty clinicians face when attempting to diagnose primary FSGS without a serum biomarker and low specificity of kidney biopsy.

## Conclusion

This study provides information on the epidemiology of a UK based population with primary FSGS: partial, complete, and combined remission rates were 23%, 49.4% and 72.4% respectively. Progression to RRT was observed in 27.6% with overall mortality also 27.6%. Partial remission was associated with reduced risk of ESKD, complete remission was associated with both reduced risk of death and progression to ESKD. This study also highlights that nephrotic syndrome may be too insensitive a phenotype to capture all primary FSGS. A subset of patients with nephrotic range proteinuria and no secondary cause identified warrant very close follow-up.

### Supplementary Information

**Additional file 1: Supplement material 1.** Raw anonymised data file of the 87 patients included within the study.

## Data Availability

Raw collected and anonymised data will be available on request. Please email Dr Thomas McDonnell Thomas.mcdonnell@nca.nhs.uk.

## Abbreviations

FSGS: Focal segmental glomerulosclerosis
uPCR: Urine protein creatinine ratios
NS: Nephrotic syndrome
UC: Undetermined cause
sAlb: Serum albumin
RAS: Renin-angiotensin system
ESKD: End-stage kidney disease
RRT: Renal replacement therapy
ACEi: Angiotensin converting enzyme inhibitors
ARBs: Angiotensin receptor blockers
EPR: Electronic patient record
KDIGO: Kidney Disease Improving Global Outcomes
CVD: Cardiovascular disease
MMF: Mycophenolate mofetil
